# Reconstruction of right ventricular outflow tract stenosis and right ventricular failure after Ross procedure – comprehensive assessment of adult congenital heart disease with four-dimensional imaging: a case report

**DOI:** 10.1186/s13256-020-02414-9

**Published:** 2020-07-23

**Authors:** Masao Takigami, Keiichi Itatani, Naohiko Nakanishi, Hiroko Morichi, Teruyasu Nishino, Shohei Miyazaki, Kosuke Nakaji, Michiyo Yamano, Yo Kajiyama, Yoshinobu Maeda, Satoaki Matoba, Hitoshi Yaku, Masaaki Yamagishi

**Affiliations:** 1 Department of Cardiovascular Medicine, Kyoto Prefectural University of Medicine, Kyoto, Japan; 2 Department of Cardiovascular Surgery, Kyoto Prefectural University of Medicine, Kyoto, Japan; 3 Cardio Flow Design Inc., Tokyo, Japan; 4 Department of Radiology, Kyoto Prefectural University of Medicine, Kyoto, Japan; 5 Department of Pediatrics, Kyoto Prefectural University of Medicine, Kyoto, Japan; 6 Department of Pediatric Cardiovascular Surgery, Kyoto Prefectural University of Medicine, Kyoto, Japan

**Keywords:** Ross procedure, 4D flow MRI, Flow energy loss, Pulmonary regurgitation, Right ventricular deterioration, Case report

## Abstract

**Background:**

Re-intervention after Ross procedure into the right ventricular outflow tract might be needed in patients in the long term. However, right ventricular outflow tract re-intervention indications are still unclear. Comprehensive assessment of total hemodynamics is needed.

**Case summary:**

A 42-year-old Japanese woman was referred to our hospital for moderately severe pulmonary regurgitation and severe tricuspid regurgitation after a Ross–Konno procedure. Thirteen years after surgery, she developed atrial fibrillation and atrial flutter and complained of dyspnea. Electrophysiological studies showed re-entry circuit around the low voltage area of the lateral wall on the right atrium. Four-dimensional flow magnetic resonance imaging revealed moderate pulmonary regurgitation, severe tricuspid regurgitation, and a dilated right ventricle. Flow energy loss in right ventricle calculated from four-dimensional flow magnetic resonance imaging was five times higher than in normal controls, suggesting an overload of the right-sided heart system. Her left ventricular ejection fraction was almost preserved. Moreover, the total left interventricular pressure difference, which shows diastolic function, revealed that her sucking force in left ventricle was preserved. After the comprehensive assessments, we performed right ventricular outflow tract reconstruction, tricuspid valve annuloplasty, and right-side Maze procedure. A permanent pacemaker with a single atrial lead was implanted 14 days postoperatively. She was discharged 27 days postoperatively. Echocardiography performed 3 months later showed that the size of the dilated right ventricle had significantly reduced.

**Discussion:**

A four-dimensional imaging tool can be useful in the decision of re-operation in patients with complex adult congenital heart disease. The optimal timing of surgery should be considered comprehensively.

## Introduction

Although the long-term results of the Ross procedure have been improving with acceptable low mortality, it is common that a re-intervention of the right ventricular outflow tract (RVOT) might be needed in the long term, in addition to treating aortic valve or aortic root [1, 2]. However, an indicator of RVOT re-intervention is still unclear due to the lack of comprehensive assessment tools of right-sided heart hemodynamics. Assessing the load of the right-sided heart system combined with the left ventricular (LV) outflow tract might be a useful way to decide on the timing of the re-intervention.

We present the case of a 42-year-old woman who underwent the Ross procedure 13 years ago. Comprehensive assessments that included four-dimensional flow magnetic resonance imaging (MRI), electrophysiology studies, and sucking force of the left ventricle measured via echocardiography, that is, interventricular pressure difference (IVPD), were quite useful in the assessment of total hemodynamics and thus a successful surgical treatment was performed.

## Case presentation

A 42-year-old Japanese woman was referred to our hospital for moderate to severe pulmonary regurgitation (PR) and severe tricuspid regurgitation (TR). Her medical history showed a coarctation repair at 9-years old and an aortic valve replacement (AVR) at 25-years old for congenital aortic stenosis. However, perioperative myocardial infarction in the anteroseptal region occurred just after the AVR, and she underwent Ross–Konno procedure using 24 mm Gore-Tex® grafting with a bulging sinus of the tricuspid valve in the RVOT position. Seventeen years after the surgery, atrial fibrillation (AF) and atrial flutter (AFL) occurred and she complained of dyspnea.

**Fig. 1:**
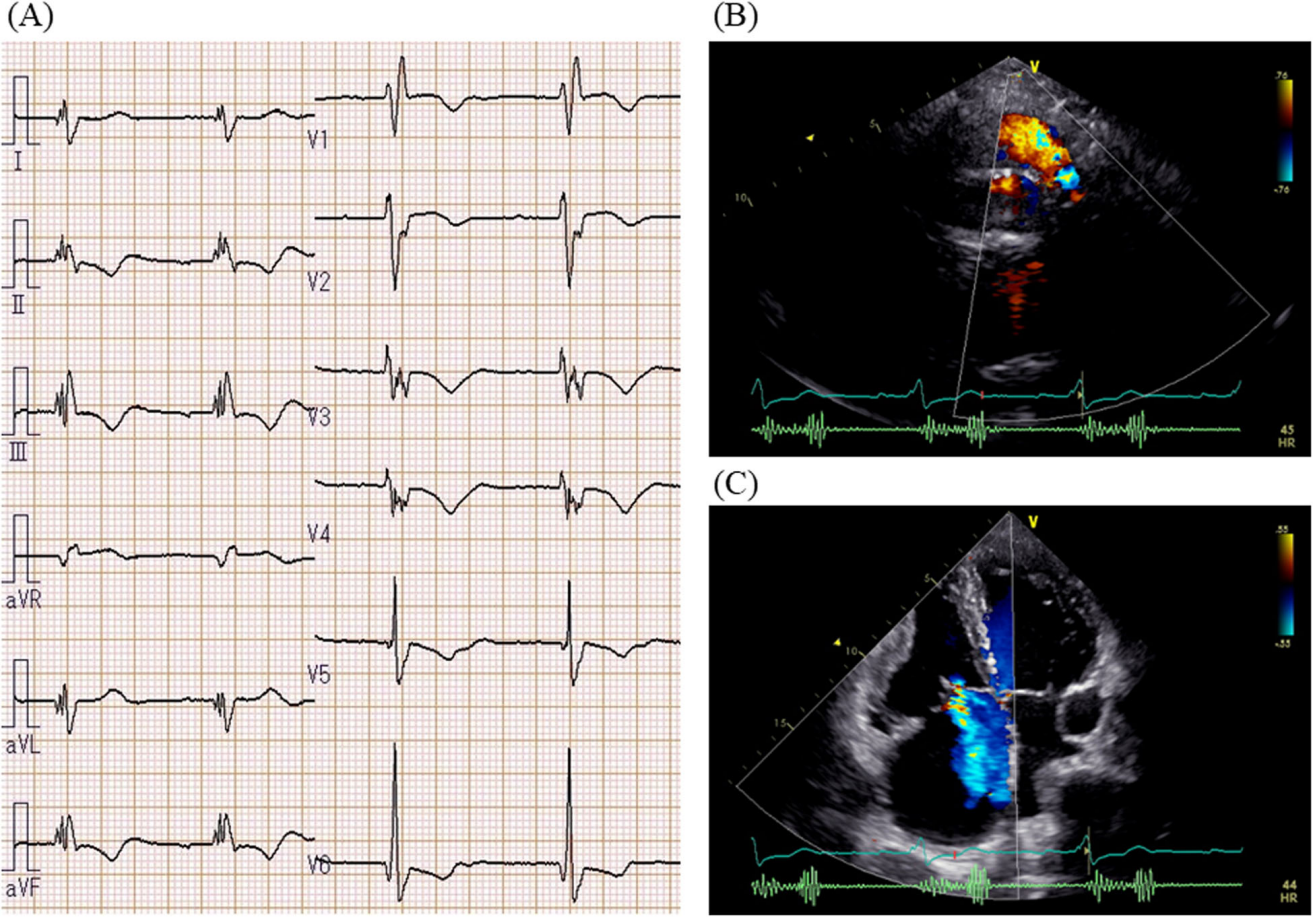
Electrocardiogram and echocardiogram. **a** Electrocardiogram showed fragmented wide QRS (167 ms) with complete right bundle branch block. **b**, **c** Echocardiogram revealed dilated right ventricular chamber and revealed moderately severe pulmonary regurgitation and tricuspid regurgitation

**Fig. 2:**
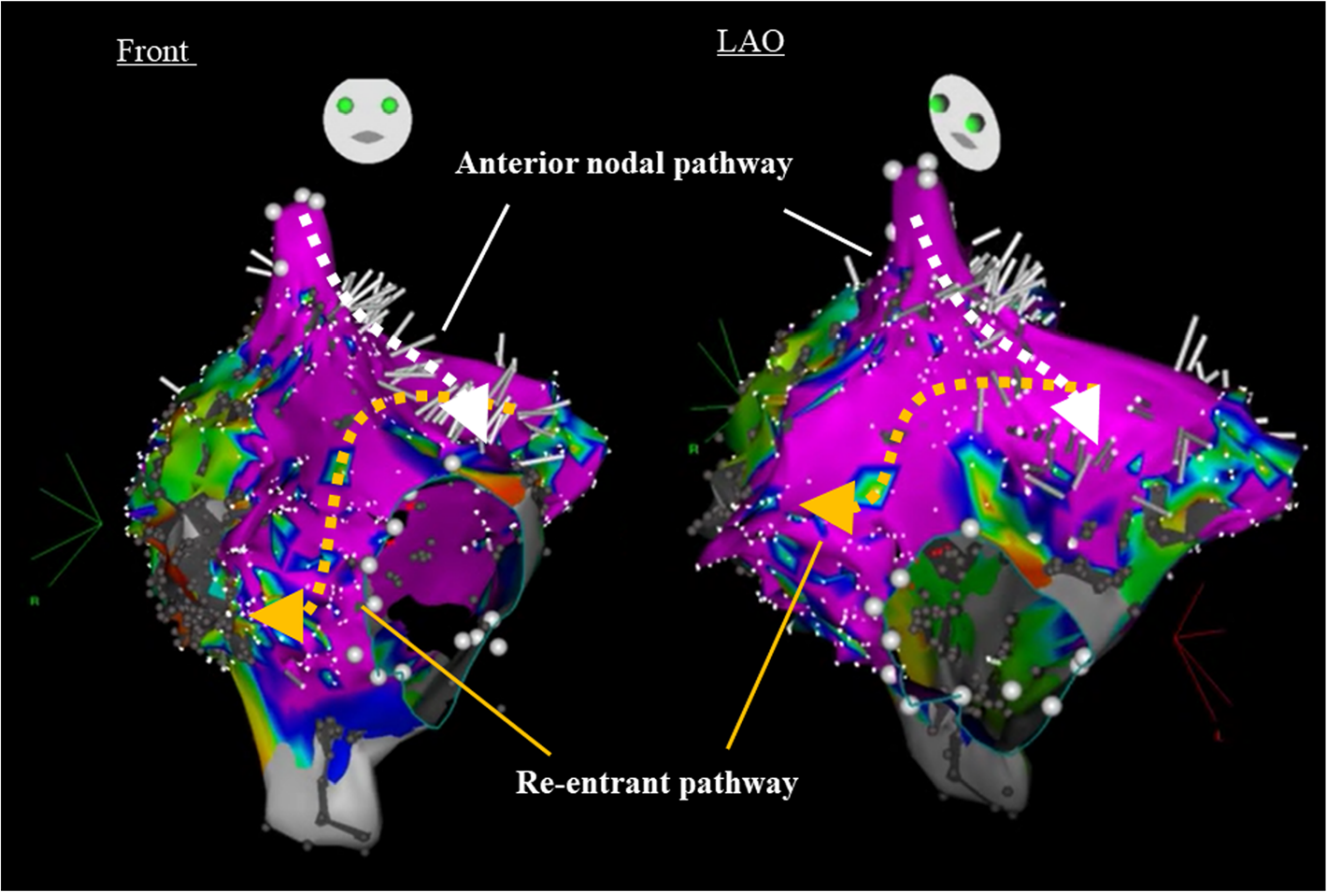
Electrophysiological study. Electrophysiological study showed re-entry circuit around the low voltage area on lateral wall of the right atrium

**Fig. 3:**
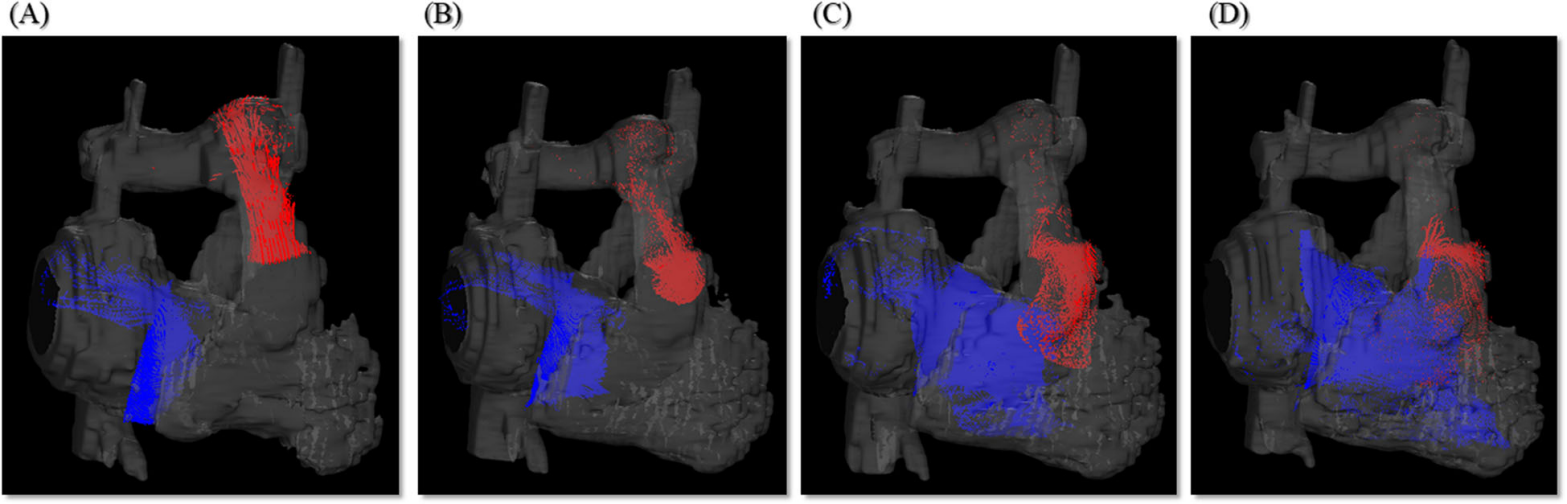
Pulmonary regurgitation and tricuspid regurgitation of three-dimensional path line from four-dimensional flow magnetic resonance imaging. **a** Early systolic phase, **b** late systolic phase, **c** early diastolic phase, and **d** late diastolic phase

**Fig. 4:**
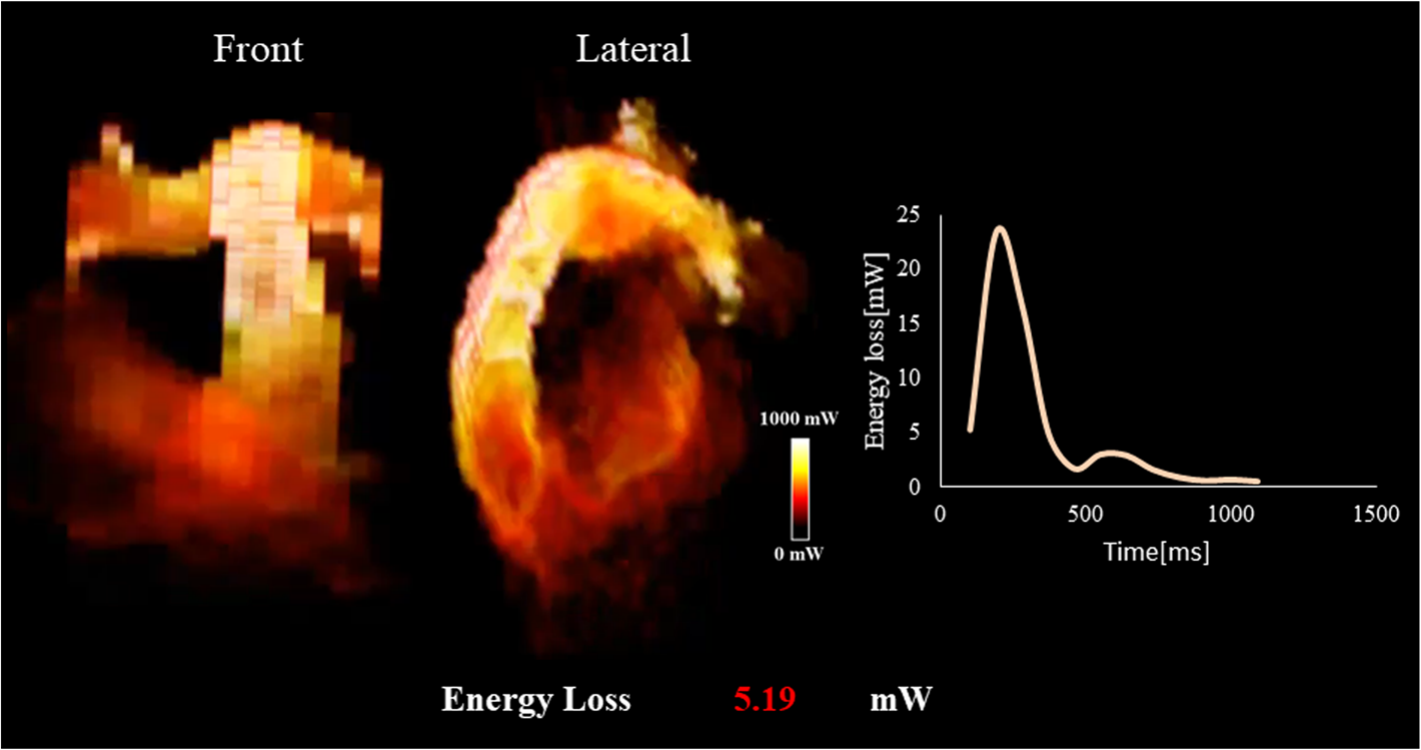
Flow energy loss. Flow energy loss in right ventricle calculated from four-dimensional flow magnetic resonance imaging was 5.19 mW, which is estimated to be five times higher than normal controls

**Fig. 5:**
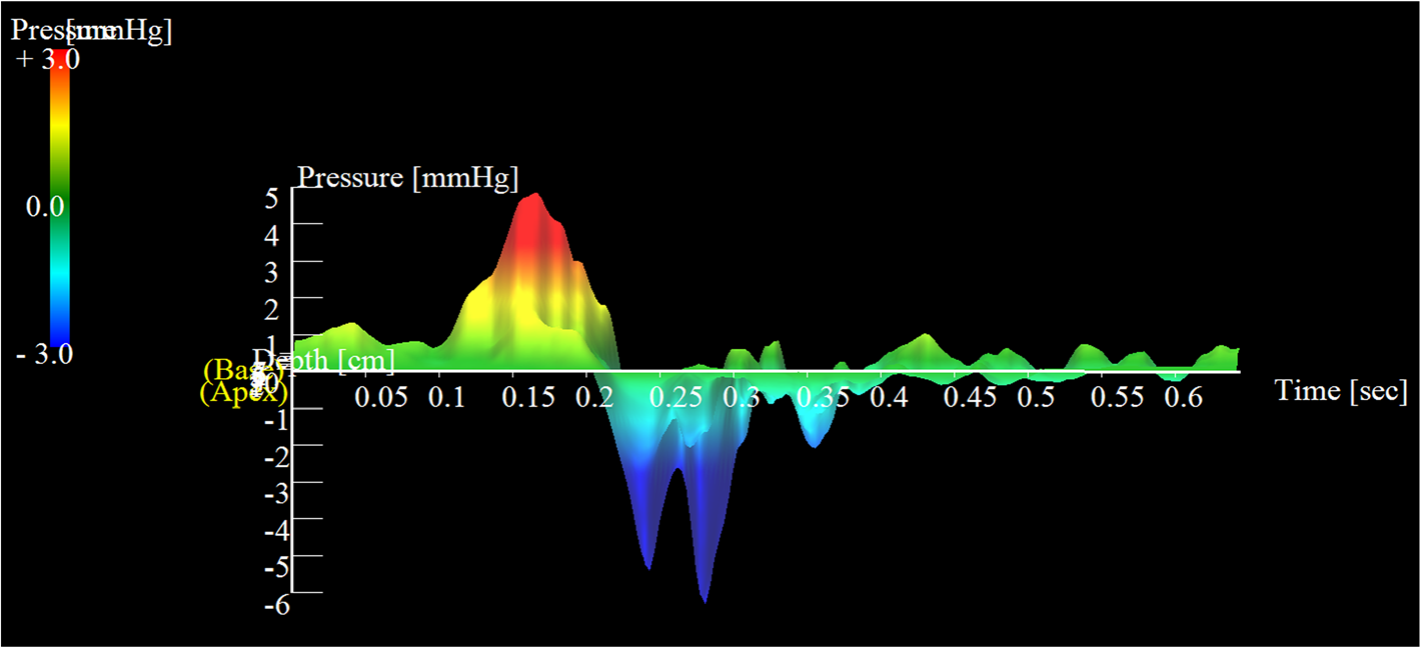
Interventricular pressure difference. Total interventricular pressure difference was 2.36 mmHg and mid-to-apical interventricular pressure difference, which was measured as two-thirds of the left ventricular length, was 1.09 mmHg

An electrocardiogram showed signs of sinus bradycardia (heart rate 48/minute), first-degree of atrioventricular block, and a fragmented wide QRS (167 ms) with complete right bundle branch block (Fig. 1a). The echocardiogram demonstrated moderately severe PR and TR and that the LV contraction was preserved regardless of hypokinesis in the septal region due to myocardial infarction (ejection fraction was 48%) (Fig. 1b and c). A three-dimensional echocardiogram measured that right ventricular end-diastolic volume index/right ventricular end-systolic volume index (RVEDVI)/(RVESVI) was 147/82 mL/m^2^. Electrophysiological studies showed the re-entry circuit to be around the low voltage area on the lateral wall of the right atrium; this re-entry leads to AFL (Fig. 2). In order to assess her right-sided heart hemodynamics in detail, we performed four-dimensional flow MRI. Four-dimensional MRI could separately evaluate PR and TR; regurgitant volumes (RVol) and regurgitant fractions (RF) of PR were calculated as 18.38 ml and 17.7%, which is estimated to be a moderate degree, and the values of TR were calculated as being 47.16 ml and 35.5%, which were estimated to be of a severe degree (Fig. 3). RVEDVI and RVESVI was 158.63/58.56 mL/m^2^, and the cardiac index (CI) was 3.18 L/minute per m^2^. Her right ventricle (RV) was slightly dilated but had not reached the point of indication for re-operation. The cardiac output was preserved. However, flow energy loss (FEL) in RV calculated from four-dimensional flow MRI was 5.19 mW, which is estimated to be five times higher than normal controls, suggesting an overload of the right-sided heart system (Fig. 4). Although she had a past history of myocardial infarction, the LV ejection fraction (LVEF) was almost preserved at 55.5%, and a thallium-201 single-photon emission computed tomography scan did not reveal any myocardial ischemia. We had to assess her LV function in detail, not only the contractions but also the diastolic function, in order to assess whether her left ventricle could receive the necessary volume from the right side of her heart after re-intervention. Total left IVPD, which was calculated based on mitral inflow measurements from color M-mode Doppler echocardiography, was 2.36 mmHg. Mid-to-apical IVPD, which was measured as two-thirds of the LV length, was 1.09 mmHg (Fig. 5). These findings show that her sucking force in left ventricle was preserved.

We decided to perform RVOT reconstruction (RVOTR), tricuspid valve annuloplasty, and right-side Maze procedure. The previous expanded polytetrafluoroethylene (ePTFE) conduit was degenerated with calcification. A new 24 mm ePTFE tricuspid valve conduit was placed in the RVOT position. Concomitantly, right-side Maze procedure was performed. The right atrium was incised via the lateral side of the atrium and ablation was carried out on the posterior and anterior walls of the atrium from the incision line to the tricuspid isthmus and to the inferior vena cava. Subsequently, a tricuspid annuloplasty was performed with Contour 3D™.

After the procedure, atrial pacing from epicardial temporary lead was needed due to sinus dysfunction and a permanent pacemaker with single atrial lead was implanted 14 days later. She was discharged 27 days postoperatively. Although we could not measure her FEL after the operation by MRI due to pacemaker implantation, echocardiography was performed 3 months later, which showed that the size of RVEDVI/RVESVI had significantly reduced to 113.5/72.7 mL/m^2^.

## Discussion

The Ross procedure is one of the standard surgical procedures for congenital aortic stenosis. Compared with aortic mechanical valve replacement, the Ross procedure has various advantages, including no need for anticoagulation medication, autograft growth, and it has excellent hemodynamics. Although the Ross procedure has an acceptable long-term survival rate of over 80% [1, 3, 4], late complications are still unsatisfactory. Nelson *et al.* showed that re-intervention in the long term is common for 87%, especially after 15 years of freedom from RVOT re-operation, which is around 53% [5]. Late complications post Ross procedure are complex due to the influence on both the left-sided and right-sided heart system and thus requires comprehensive evaluation of both the right-sided and left-sided heart system. In this case, we had several difficulties to determine the operative indications: moderate PR, moderate pulmonary stenosis (PS), severe TR, atrial tachycardia, sinus dysfunction, and LV systolic and diastolic functions.

We comprehensively assessed cardiac workload and problems in her right-sided heart failure. FEL is known as a parameter of cardiac workload to predict ventricular deterioration in various heart diseases [6, 7], including heart valve disease, cardiomyopathy, and congenital heart disease. Regarding the right ventricular deterioration, Shibata *et al*. reported that FEL through the pulmonary valve after tetralogy of Fallot (TOF) repair was correlated with QRS duration prolongation in an electrocardiogram [8]. In our case, her FEL of the right-sided heart system calculated from four-dimensional flow MRI was very high due to the combination of moderate RVOT stenosis, moderate PR, and severe TR. It is reported that RV cannot obtain reverse remodeling of the normal range after pulmonary valve replacement (PVR), if re-operation is too late [9]. Integrated FEL in the right-sided heart system might be a useful tool to consider the timing of re-operation before RV dysfunction or enlargement is developed.

IVPD represents left ventricle sucking force, which has a good correlation with tau index used as the gold-standard indicator of diastolic function [10, 11]. Mid-to-apical IVPD shows an indicator of active sucking force [12, 13]. Surgical intervention of the tricuspid and pulmonary valve regurgitation increased the LV preload; therefore, the surgical indication should be carefully considered for each patient with an impaired LV function. Her total and mid-to-apical IVPD showed that her sucking forces were preserved, suggesting a good tolerance against LV volume increase after re-operation.

Arrhythmias and conduction disturbances are also common after late complications of a repaired Ross procedure. Atrial tachy-arrhythmias result in LV dysfunction, leading to systolic heart failure [14, 15]. Some reports have shown that catheter ablation for AF improved LV function and the probability of survival [16, 17]; however, atrial arrhythmic involvement in RV dysfunction has not really been explored. In this case, ablation for AFL might have been effective to improve the RV dysfunction after RVOTR. Furthermore, evaluation of this using Carto® mapping enabled appropriate ablation. If atrial tachycardia had continued after the operation, ablation of atrioventricular node would have been needed to keep an appropriate heart rate. Although, in fact, she underwent a pacemaker implantation due to her sinus dysfunction, only one lead in the atrium was required after the operation. We could avoid the placement of a ventricular lead through the tricuspid valve, owing to the disappearance of her arrhythmia. Because patients with adult congenital heart disease (ACHD) have often undergone several previous surgeries and their hearts are anatomically different from normal hearts, the cause of arrhythmia is often complicated. Electrical mapping before re-operation using Carto® mapping is useful to treat arrhythmia in patients with ACHD.

## Conclusions

A four-dimensional imaging tool was useful in making the decision of re-operation in a patient with complex ACHD. We should consider comprehensively the optimal timing of surgery.

## Data Availability

Not applicable.
